# Prevalence of Smoking Among School Students in Iraq

**DOI:** 10.7759/cureus.67048

**Published:** 2024-08-16

**Authors:** Omaima A Zubair

**Affiliations:** 1 Family and Community Medicine Department, College of Medicine, University of Mosul, Mosul, IRQ

**Keywords:** adolescent addiction, peer pressure, secondary school students, shisha smoker, smoking tobacco

## Abstract

Background

Developing communities like Iraq are critical to building a good community environment. Many complex patterns of behaviors like smoking among adolescents have been exacerbated rapidly in the current era which led to changes in the Iraqi community’s perspectives and life expectancy. This study aims to find out the prevalence of smoking among secondary school students in Iraq and their perceived contributing factors.

Methods

A cross-sectional study design was used with a representative sample of Iraqi school students recruited through a multistage cluster randomization from the Nineveh Governorate’s intermediate and secondary schools to ensure the representation of the general public strata. A total of 330 students were randomly selected from eight schools distributed all over Mosul city (the center of Nineveh Governorate) and its boundaries semi-rural areas. Data collection utilized a standardized, anonymous questionnaire based on the Global Youth Tobacco Survey (GYTS) administered within classrooms with the researcher’s presence. The questionnaire included questions related to sociodemographic characteristics of the students, smoking state, smoking types, frequency of smoking, relative smoking state, opinion on predisposing factors for initiation of smoking, and knowledge about hazards of smoking. Data analysis was done using SPSS version 26 to calculate frequency distribution with further logistic regression analysis was performed to identify statistically significant factors contributing to initiation of smoking among the student participants with p-value estimation of any compares considered significant below 0.05.

Results

Prevenance of all types of smoking was 24.5% from 330 students with 30% from 246 males and 7% from 84 females has been encountered. Hookah (Shisha) was the most prevalent type of smoking. About a third of the smoker sample reported smoking at least once daily. Peer pressure (OR=3.49, P< 0.001) or family smoking (OR=1.769, P=0.019) emerged as the strongest influence for initiation of smoking, besides stress (OR=2.23, P= 0.04) and personality traits like stammering and jealousy (OR=2.58, P= 0.013), (OR=2.22, P= 0.017, respectively) have also significant odds. Interestingly, media (movie star) influence played a significant role also. (OR=1.492, P= 0.045).

Conclusion

The prevalence of smoking among the study sample was 24.5 % out of 330 participating students. Significant influencing factors were found that raised the concern and necessitated the development of targeted interventions. Implementing evidence-based strategies, such as comprehensive school-based educational programs and smoke-free indoor air policies, can significantly improve public health outcomes.

## Introduction

Smoking initiation is intense among young people which strongly influences future smoking prevalence. Remodeling adolescents’ habits will lower the prevalence of adult tobacco use later and maintain sustainability. The average age of initiation of smoking was significantly decreased and adolescents are the most vulnerable age [1]. Surveillance of tobacco use among adolescents helps set priorities in developing tobacco control policies [2]. Most of the preventable premature deaths worldwide are related to tobacco smoking which account for more than 8 million deaths and cost US$ 1.4 trillion from the global economy each year. [3]. Recently, the popularity of waterpipe smoking has increased globally. Despite much evidence regarding the harmful effects of waterpipe smoking, the common belief in the community it has lesser harmful effects than traditional cigarettes [4].

The physical and psychological development of adolescents can be greatly influenced by smoking, even the possibility of asthma development and cardiovascular diseases is greater among regular adolescent smokers than that in nonsmokers [5].

It was indicated by research that nicotine, found in cigarettes and other tobacco products, affects the development of the adolescent brain, especially areas involved in reward and pleasure. This makes adolescents more susceptible to engaging in more risky deviant behavior of drugs and alcohol [6]. In Iraq, conflicts have been predominant in the last 20 years due to repeated war and ISIS invasions which create post-traumatic disorder that encourages deviation from normal behavior and smoking is regarded as one of these deviations [7]. The continuous absence of legitimacy to restrict smoking in restaurants or cafés in Iraq contributes to smoking dissemination leading to the tobacco epidemic. Trivial efforts were presented to restrict public smoking in large malls or restriction in restaurants but it only applied in limited areas which didn’t reach the goal of the smoking cessation program that was announced ten years ago.

Besides, fun and recreational scientific or sports areas are very limited in Iraq and all business leaders are directed toward opening café or restaurants which attract a lot of young people who spend several hours playing dominos and other video games which allow for rapid profit. No real use of these men's powers or proper direction toward building or development of the community, instead they are drawn toward the community and peer pressure influence without proper family guidance. Worldwide, children getting farther from their families with expanding technology among children's hands and increasing life responsibilities of the parents lead to decreased attention and follow-up to their children regarding what they do in their spare time [8]. This study aims to determine the current smoking prevalence among a sample of secondary school students in Iraq and to determine the common influencing risk factors.

## Materials and methods

A cross-sectional survey was employed; it was carried out using a multistage cluster sampling design to recruit secondary school adolescents from the Nineveh Governorate which is located in Iraq that included urban areas (left and right bank of the city) as well as semi-rural areas located outside the Mosul city boundaries. The inclusion criteria for participants included those students who enrolled as intermediate or secondary school students in Nineveh Governorate who attended the school on the day of the survey and their age between 12 and 20 years of age. The exclusion criteria were any students who were absent on the day of data collection or those who were unable to complete the questionnaire for different reasons such as exams or sickness. The sample size was calculated using an acceptable error level of 5%, an expected population proportion of smoking of 0.21, and a 5% type I error rate.

The total number of students all over Nineveh Governorate which was provided by the Directorate of Education in Nineveh was 202,882 students for the year 2024. By applying the equation of estimation of sample size estimation, the resulting sample size estimated is about 254 as follows:

n= N.Z2.P.(1-P)/E2.(N-1)+Z2 .P.(P-1)

where

n= is the needed sample size, N= actual population size (202882), Z= Z-score calculated as (1.96 for 95% confidence level), P is the estimated prevalence calculated as 21% from the previous studies and E is the margin of error which is estimated to be 5%.

To avoid any possible dropout of the sample or incomplete questionnaire and to increase the accuracy and reliability of the data, the researcher intended to select a larger sample size than the calculated number within the available resources and time.

A total of 330 students were randomly selected from eight schools, exceeding the required sample size. The multistage cluster sampling technique employed included the following stages: Stage 1) Random selection of eight schools from both urban and semi-rural areas of Nineveh Governorate, ensuring male-to-female representation of the sample; stage 2) Random selection of students within the selected schools from the school’s classes; stage 3) Random selection of the students from the single class where every other 3rd student was chosen.

The data was collected using a self-administered questionnaire adapted from the Global Youth Tobacco Survey (GYTS) [9]. The questionnaire was completed in the classroom in the presence of researchers. The questionnaire included sociodemographic characteristics like age, gender, grade, location, parental education and occupation, family size, etc., smoking status (Ever or current smoker, and never smoked), Smoking behaviors (how often they smoke, type of tobacco products used and exposed to second-hand smoking or nearby smoking), opinion on predisposing factors for initiation of smoking, and knowledge about hazards of smoking.

The researcher collected data in classrooms. The process was voluntary and confidential as personal details were not included.

Ethical considerations

This study was started by obtaining the administrative agreement from the Medical Research Ethics Committee (MREC) with reference no. UOM/COM/MREC/22-23 (34) followed by approval from the statistical department in the planning division at the Directorate General of Education, Nineveh Governorate. At the beginning of each school visit, the principals provided written consent to allow data collection. The anonymity of participants was ensured by excluding identifiers like names, addresses, and phone numbers from the data collection tool. Participation was entirely voluntary, and no incentives were offered while respondents’ students provided theirs willing to participate.

Data analysis

Microsoft Excel facilitated data entry which it later cleaned. SPSS version 26 helped with statistical analysis. Descriptive statistics included frequency and percentage distributions whenever needed. Inferential statistics for smoking status was treated as a dependent variable and the independent variables included relevant sociodemographic factors such as behavioral and attitudinal factors. For this reason, a logistic regression model for current smoking risk factors was calculated using relevant sociodemographic data such as relevant sociodemographic as well as behavioral and attitudinal variables were introduced first into the equation one after the other.

To measure the strengths of association between each factor considered individually an odds ratio (OR) was computed with a significant p-value considered < 0.05.

## Results

**Figure 1:**
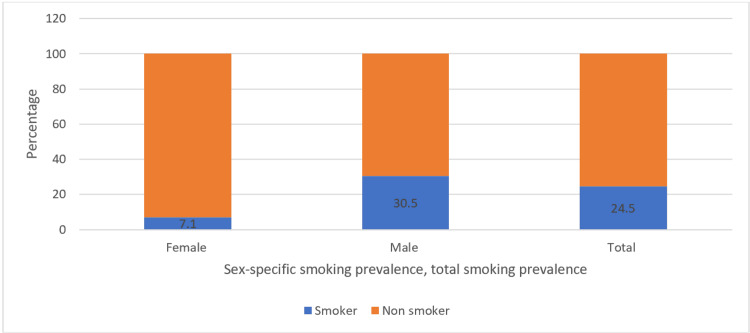
Prevalence of smoking among study sample students (N= 330) Descriptive statistics are used for the calculation of total numbers and frequencies. The data are represented as the percentage (%) of smokers among the total female student participants (N=84), the total male student participants (N=246), and the total student participants (N=330), respectively.

The prevalence of smoking is illustrated in (Figure 1) which shows that 24.5% of the 330 participants were smokers of either or both types of smoking. The prevalence of smoking among males is higher than among females with 30.5% of 246 males and 7.1% of 84 females.

**Figure 2:**
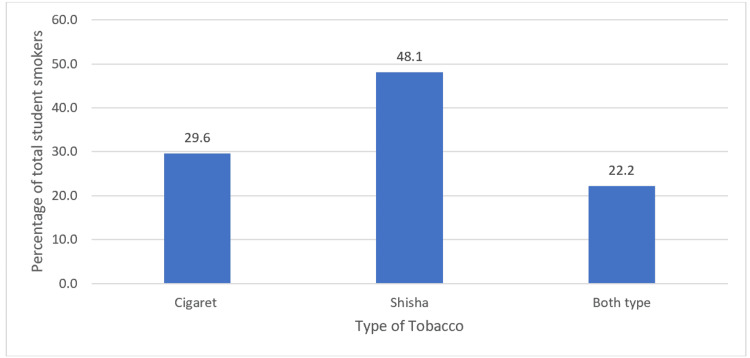
Types of smoking among smoker students Descriptive statistics are used for the calculation of total numbers and frequencies. The data are represented as the percentage of each type of smoking among the 81 smoker student participants (N=81)

Figure 2 demonstrated that Shisha was the most prevalent type of smoking, as about half of the 81 smoker participants preferred Shisha for smoking, added to them those who smoked both Shisha and cigarette smoking which presented 22% of 81 smoker students.

**Figure 3:**
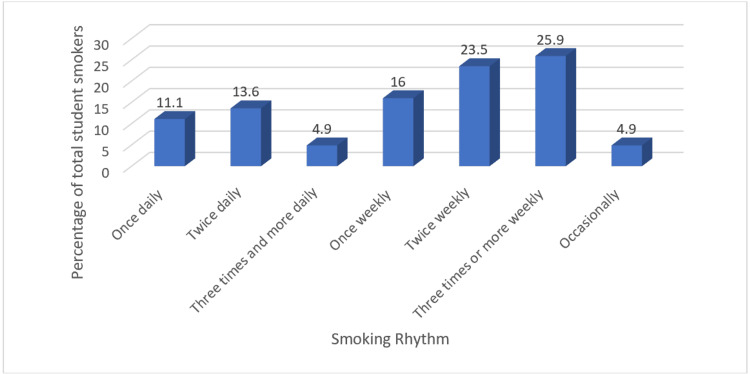
Smoking rhythm among smoker participants Descriptive statistics are used for the calculation of total numbers and frequencies. The data are represented as the percentage of each smoking rhythm among smoker student participants (N=81)

Figure 3 shows that about a third of the 81 smoker participants reported smoking at least once daily as considered a collective result for once, twice, or three times a day.

Table 1 shows that 74.5 % out of 330 participants were males. Those aged 16-18 years old were presented in 67.2% of the study participants. Accordingly, 68.7% were from high-grade classes. Those living inside the city presented in 62.1 of the sample. Employed fathers were 57.8 % of the participants while mothers presented 61.1% as housewives. More than half of the participants reported having a good living level (54.8%), and 36.6% lived with more than one family at home.

**Table 1:** Sociodemographic characteristics of students participants *Descriptive statistics of number, and % were used for calculation of frequency out of the N=330 participants.

Variables	No. (%)* N=330
Sex	
Male	246 (74.5)
Female	84 (25.4)
Age	
12-15	97(29.3)
16-18	222(67.2)
19-20	11 (3.3)
Grade	
Intermediate grades	103 (31.2)
High grades	227 (68.7)
Residence	
Inside city (public school)	205 (62.1)
Outside city (rural, semi-rural)	125 (37.8)
Father’s job	
Employed	191(57.8)
Unemployed (free or private job)	139 (42.1)
Mother’s job	
Housewife	203 (61.5)
Employed	127 (38.4)
Levels of living	
Poor	33 (10)
Medium	102 (30.9)
Good	181 (54.8)
Excellent	14 (4.2)
Number of families in one home	
One family	209 (63.3)
More than one family	121 (36.6)

Table 2 presents the answers of students to different questions related to their knowledge and attitudes about smoking, comparing smokers (N=81) and nonsmokers (N=249). The data reveals that nonsmokers are significantly more aware of the side effects of smoking (61%) compared to smokers (45%), with a p-value of 0.0026 indicating statistical significance. Advice from family to quit smoking is reported by 70.03% of smokers and 79.5% of nonsmokers, though the difference is not statistically significant (p=0.23). Similarly, school advice shows a higher, but not statistically significant, p-value equal to 0.06. The influence of media on quitting smoking is noted by 14.8% of smokers and 8.03% of nonsmokers (p=0.25). The intent to quit smoking is expressed by 23.4% of smokers, but the corresponding data for nonsmokers is inapplicable.

**Table 2:** The answers of students regarding their information about smoking *The Chi-square test of the 2×2 table was used for analysis with the p-value considered significant < 0.05 to indicate the statistical significance of differences between the two groups.

Factors	Those who answer yes among smokers (N=81)	Those who answer yes among nonsmokers (N=249)	P-value
Do you know the side effects of smoking?	37 (45 %)	152 (61%)	0.0026
Do you receive advice from family to quit smoking?	57 (70.0%)	198 (79.5%)	0.23
Do you receive advice from school to quit smoking?	41(50.6%)	92 (36.1%)	0.06
Does the media have influence on quitting smoking?	12 (14.8%)	20 (8.0%)	0.25
Do you want to quit smoking?	19 (23.4%)	-	-

Table 3 presents the smoking status of students’ relatives smoking status that smoker students have more exposure to smoking relatives than nonsmoker students 61.7 % of 81 smokers versus 47.3 % among 249 nonsmoker students. Descriptive frequencies were used to present the data.

**Table 3:** Smoking state of students’ relatives *Descriptive statistics of number, % were used for calculation of frequency out of the N=330 participants.

Smoking state of students	Relative smoking state		Total
	Relative is smoker	Relative is not a smoker	
Smoker (% within smoking state)	50 (61.7)	31 (38.2%)	81 (24.5%)
Nonsmoker (% within smoking state)	118 (47.3%)	131(52.6 %)	249 (75.45%)
Total	168 (50.9%)	162(49.1%)	330 (100.0%)

Table 4 represents a comprehensive overview of the factors influencing smoking habits among participants, focusing on the importance of social and psychological variables in smoking initiation. The logistic regression analysis identifies significant predictors of smoking initiation among participants. Being male is associated with a significantly higher likelihood of initiating smoking (OR = 3.004, p < 0.001). Friends’ pressure and the effects of stress are strong predictors as OR = 3.49, p < 0.001, and OR = 2.23, p = 0.04, respectively. Additionally, stammering (OR = 2.58, p = 0.013) and jealousy or imitation (OR = 2.22, p = 0.017), show significant effects on initiation of smoking. The smoking status of relatives (OR = 1.769, p = 0.019), and media influence (OR = 1.492, p = 0.045) are also significant factors. While lower socioeconomic status and residence inside the city show increased odds for smoking initiation, these variables are not statistically significant at the conventional levels (p < 0.05).

**Table 4:** Logistic regression of variables that could be related to smoking habit initiation among participants The analysis was conducted using a binary logistic regression model, which calculates the ORs. The p-values indicate the statistical significance of each factor in predicting smoking initiation were considered significant < 0.05. The logistic regression analysis was conducted using SPSS statistical software, with a significance level set < 0.05.

Variable	Coefficient (β)	Odds Ratio (e^β)	P-value
Sex (Male)	1.100	3.004	<0.001
Mother’s job (Employed)	0.450	1.568	0.137
Father’s job (Employed)	-0.200	0.819	0.529
Socioeconomic Status (Ref: Middle)			
Bellow middle	0.630	1.878	0.056
Good	-0.250	0.779	0.42
Excellent	-0.45	0.63	0.245
Residence inside city	0.520	1.68	0.080
Incentive to smoking (Personal desire)			
Friends pressure	1.250	3.49	<0.001
Community pressure	0.600	1.82	0.059
Stress effect	0.8	2.23	0.04
Stammering	0.950	2.58	0.013
Jealousy (imitation)	0.800	2.22	0.017
Relative’s smoking status	0.570	1.769	0.019
Media effect	0.400	1.492	0.045

## Discussion

Adolescence is a risky period that witnesses the initiation of many risky habits, as most smokers start smoking regularly before the age of 20. This study focused on the increasing phenomena of smoking among secondary and high school students accompanied by a decrease in the age of smoking initiation. Recently, after political stability in Iraq, limited research focusing on this issue and the epidemic of smoking is waiving on the horizon.

The study shows that the total current prevalence of smoking was 24.5% out of 330 students, with 30% out of 246, and 7% out of 84 among male, and female participants, respectively. This represents an increase of 3% from the 21% out of 1750 participants of the GYTS which was done in 2012 including adolescents from all of Iraq with tobacco as the most prevailing type of smoking over Shisha at that time [10], this figure was replaced by Shisha over a cigarette in the current study. This figure may be doubled or tripled for those of non-educated youth strata. A study done in Karbala Iraqi governorate in 2023 found the prevalence of smoking was 25.3% among secondary school students [11]. Moreover, A study done in Duhok in 2022 found that 36% out of their 420 secondary school male students aged 15-18 enrolled in the study were smokers [12]. Current and concomitant studies revealed an increasing percentage of smokers who anticipated epidemic dissemination of smoking if no fast suitable restrictive measures are implicated.

Besides, the current study revealed the prevalence of smoking of 7% out of 141 females in the study sample. Although this figure shows decreasing frequency from reports of the GYTS which indicated that 12.7% of their sample size of adolescent girls aged 13-15 were smokers [10]. This decrease is only registered in Mosul, however, it might be duplicated or tripled in Baghdad or in the Kurdistan region in which their girls have more freedom to go to cafés to smoke with their friends. The logistic regression analysis of predicting risk factors for smoking shows that being male, those with stammering personalities, and those who like to imitate (jealousy) are strong predictors as stimulating factors for smoking initiation. This appeared from a significant increase in odds among those affected by their friends’ pressure, relative smokers, and media effects like movie stars. Even though community pressure raises the odds of smoking by 1.82 times, it is marginally significant. More than half of the present participants (smokers and nonsmokers) reported the presence of close relatives smoking near them without curtain which led to adolescents’ exposure to smoking even if they were not smokers. The Centers for Disease Control and Prevention (CDC) reported that adolescents who lived with smoker members of families are more likely to be smokers because of their willingness to imitate and the prevalence of social norms [13]. Many types of research indicated troubles with passive smokers as a predisposing risk factor for allergies or other diseases among children and youth [14]. The smoking prevalence varies between different regions of the world, as it has decreased in high-income countries, but low- and middle-income countries are still at risk [3]. Yet, tobacco control in the Arab world has common patterns and shared challenges that need to be specified to address its cause.

Male patterns of smoking, water pipes, and lack of effective tobacco control programs are the major issues to be focused on [15]. Recently, the prevalence of ever smokers among secondary school students in the United Arab Emirates was 39 % among 560 participants enrolled in their cross-sectional survey performed in Ajman city [16], and a study of students’ smoking status in Saudi Arabia who included the secondary school found that 37% from 695 respondents students were currently smokers and from these, 83.7% had started smoking at the age of 14 years or less. Family influence was the most common reason for smoking, especially the presence of someone at home who smoked or their friends who smoked [17]. Similarly, other studies supported the same influencing factors [18,19].

The 2023 data from the CDC shows 4.0% of middle school and 13.4% of high school students in the USA from their national yearly survey reported current use of a tobacco product [20].

Iraq has become a part of the World Health Organization (WHO) Framework Convention on Tobacco Control (FCTC)since 2008 [21]. The Ministry of Health in Iraq launched, more than 10 years ago, a Law No. 19 of 2012 on Combating Smoking, however, the implementation facing difficulties and failure [22]. The Ministry of Health made cooperation with the Ministry of Education, which expanded to include all governorates,” and “the project was launched under the name Anti-Smoking Schools.”

The smoking habit initiation is a multifaced factor including, sociodemographic, behavioral, and environmental or social influences. Besides the psychological factors and personal knowledge and attitude. These factors react together to create an environment that leads to the initiation of smoking. This perspective needs to increase public awareness to enhance the family's effect on positive health behavior rather than negatively destroying behavior. The analysis of current data shows that being male gender, living in urban regions, stammering personality, and those who like to imitate have a significantly increased risk for initiation of smoking. Several studies supported the increased risk of smoking related to those factors. Adolescents who live in the urban region are more likely to smoke than those in the countryside or rural regions [23]. The behavioral effect appeared through peer pressure and household smoking effect on the initiation of smoking habit as they carry significantly increased risk. Similarly, the effect of friends and family was reported as a strong predisposing factor to initiate smoking in adolescents [24].

Stress is the most common cause chosen by the participants to have an effect on smoking behavior. Sometimes students use smoking as a coping mechanism to deal with psychological upset as wrongly believe that smoking provides stress relief [25], and in Iraq, there is cultural acceptance of smoking especially among males. This together with the availability of different types of smoking contributes to the increasing rate of smoking [26]. Sometimes the incentives come involuntary from the media, when looking at movie stars with frequent exposure to smoking are seen encouraging imitation of the behavior [27]. The current analysis revealed increased odds of media exposure. There was no significant odds effect found among different social groups neither regarding parental employment status as governmental employment mostly indicated the education state. Other studies reported that low social class could exert a risk for initiating smoking [28].

The main drawback of the current study is the limited sample size of one governorate because of limited resources which could affect the generalizability of the results another limitation is the non-specification of E-smoker as it was included within cigarette smoking. The current study was focused only on tobacco smoking risky adolescent behavior, however, a further study involving a national survey to explore other Iraqi adolescent risky behaviors like drugs, alcohol, sedentary lifestyle, and bullying should be done. This will open a window for implementing prevention programs targeting young people and preventing suffering while saving millions of lives.

## Conclusions

The concerning rise in smoking prevalence among Iraqi secondary school students necessitates the performance of the National Youth Tobacco Survey and the development of targeted interventions. Implementing evidence-based strategies, such as comprehensive school-based educational programs and smoke-free indoor air policies, can significantly improve public health outcomes. These interventions can empower Iraqi youth to make informed decisions about their health, leading to a longer and healthier life expectancy for all citizens. Besides, it’s needed to use media as an instrument to reshape the community in a good manner.
